# Atezolizumab in locally advanced or metastatic urothelial cancer: a pooled analysis from the Spanish patients of the IMvigor 210 cohort 2 and 211 studies

**DOI:** 10.1007/s12094-020-02482-9

**Published:** 2020-09-08

**Authors:** M. Sotelo, T. Alonso-Gordoa, P. Gajate, E. Gallardo, R. Morales-Barrera, J. L. Pérez-Gracia, J. Puente, P. Sánchez, D. Castellano, I. Durán

**Affiliations:** 1 Marqués de Valdecilla University Hospital, Edificio Sur. Despacho 277, Avda Valdecilla s/n, 39005 Santander, Spain; 2 Ramon y Cajal University Hospital, Madrid, Spain; 3 Parc Taulí Hospital Universitari, Institut d’Investigació i Innovació Parc Taulí I3PT, Universitat Autònoma de Barcelona, Sabadell, Spain; 4 Vall d’Hebron University Hospital, Barcelona, Spain; 5 University Clinic of Navarra, Pamplona, Spain; 6 Medical Oncology Department, Hospital Clínico San Carlos, Instituto de Investigación Sanitaria del Hospital Clínico San Carlos (IdISSC), CIBERONC, Madrid, Spain; 7 Medical Department, Roche Farma S.A., Madrid, Spain; 8 Doce de Octubre University Hospital, Madrid, Spain; 9 Instituto de Investigación Marqués de Valdecilla (IDIVAL), Santander, Spain

**Keywords:** Atezolizumab, Metastatic urothelial cancer, Duration of response, Spain

## Abstract

**Background:**

The studies IMvigor 210 cohort 2 and IMvigor211 evaluated the efficacy of atezolizumab in patients with locally advanced or metastatic urothelial cancer (mUC) upon progression to platinum-based chemotherapy worldwide. Yet, the real impact of this drug in specific geographical regions is unknown.

**Materials and methods:**

We combined individual-level data from the 131 patients recruited in Spain from IMvigor210 cohort 2 and IMvigor211 in a pooled analysis. Efficacy and safety outcomes were assessed in the overall study population and according to PD-L1 expression on tumour-infiltrating immune cells.

**Results:**

Full data were available for 127 patients; 74 (58%) received atezolizumab and 53 (42%) chemotherapy. Atezolizumab patients had a numerically superior median overall survival although not reaching statistical significance (9.2 months vs 7.7 months). No statistically significant differences between arms were observed in overall response rates (20.3% vs 37.0%) or progression-free survival (2.1 months vs 5.3 months). Nonetheless, median duration of response was superior for the immunotherapy arm (non-reached vs 6.4 months; *p* = 0.005). Additionally, among the responders, the 12-month survival rates seemed to favour atezolizumab (66.7% vs 19.9%). When efficacy was analyzed based on PD-L1 expression status, no significant differences were found. Treatment-related adverse events of any grade occurred more frequently in the chemotherapy arm [46/57 (81%) vs 44/74 (59%)].

**Conclusion:**

Patients who achieved an objective response on atezolizumab presented a longer median duration of response and numerically superior 12 month survival rates when compared with chemotherapy responders along with a more favorable safety profile. PD-L1 expression did not discriminate patients who might benefit from atezolizumab.

## Introduction

Urothelial cancer (UC) is a frequent disease globally with over 549,000 new diagnoses worldwide in 2018 [1]. The incidence and mortality of UC differ across countries due to differences in risk factors, diagnostic practices and accessibility of therapies. In Spain, the incidence of this tumour is particularly high, ranking fifth altogether and rising up to the fourth most frequent malignancy among males. In 2020, more than 22,000 diagnoses of UC are expected in our country representing a relevant medical challenge [2]. Around 70% of UC diagnoses are in early stages (non-muscle-invasive disease) where prognosis after appropriate treatment remains overall favourable with a 5 year overall survival (OS) exceeding 90%. Nevertheless, 25% of patients present with disease invading the *Muscularis propria* or beyond (muscle-invasive UC) and despite optimal management, roughly 40–50% will relapse, presenting metastatic disease not amenable for surgical treatment. Additionally, about 5% of the new diagnoses present with advanced disease at diagnosis. Metastatic UC harbours a poor prognosis with a 5 year OS that historically does not exceed 10–15% [3, 4]. Chemotherapy regimens based on cisplatin have become the most utilized ones in this setting. The cisplatin–gemcitabine (CG) combination provides objective response rates (ORR) in the range of 50% with 12% of complete responses (CR) and a median OS of around 15 months [5]. Despite these promising results, two facts have historically limited progress in advanced UC therapeutics. First, about 50% of patients with metastatic disease are considered unfit to receive cisplatin according to predefined criteria (the Galsky’s criteria). These patients can only receive alternative regimens, such as carboplatin, or other regimes with inferior outcomes to cisplatin [6, 7]. Second, nearly all patients with metastatic disease, regardless of their response to first-line treatment, will end up progressing and the classic chemotherapy agents explored in the second line have historically provided scarce benefit with an ORR of less than 10%, a short duration of response (DoR) and a median OS of about 7 months [8]. Therefore, defining a better treatment for cisplatin-unfit patients and improving poor outcomes in the second-line setting are unmet needs.

In this context, a different approach to treat UC has been developed considering specific features of this tumour [9]. One distinctive characteristic of UC is the high rate of somatic mutations observed that may potentially increase the capacity of the host immune system to distinguish UC cells as foreign. This would eventually lead to proper tumour cell identification and elimination [10]. Nevertheless, UC cells might escape immune surveillance through the expression of programmed death-ligand 1 (PD-L1) in the tumour microenvironment [11–13]. Back in 2014, an expansion cohort of about seventy patients with heavily pretreated advanced UC was treated with an anti-PD-L1 antibody (i.e. atezolizumab) as part of a multi-tumour phase I study. Beyond positive safety data, unprecedented activity was reported with ORR in the range of 40–50% in patients whose tumours highly expressed PD-L1-positive tumour-infiltrating immune cells [14]. These results led to considering testing this compound in advanced UC patients beyond platinum progression and, due to the favourable toxicity profile, this drug appeared as an option for cisplatin-unfit patients. Hence, two separate trials (IMvigor 210 and IMvigor 211) were launched and tested atezolizumab in different populations. IMvigor 210 was a phase 2 study with two single-arm cohorts where patients either treatment naïve unfit for cisplatin (cohort 1) or with progression beyond first-line chemotherapy (cohort 2) received atezolizumab with the ORR as the primary endpoint. The positive results of these two cohorts moved atezolizumab development forward and led to drug approval and the incorporation of this compound in treatment algorithms [15, 16]. Across drug development, the expression of PD-L1 appeared as a potential positive predictor of response to immunotherapy. Atezolizumab was subsequently tested in a randomized phase 3 study (IMvigor 211) that compared chemotherapy vs. atezolizumab in patients with advanced UC and progression beyond platinum-based chemotherapy. The primary endpoint of OS was tested hierarchically in pre-specified populations according to the PD-L1 expression assuming greater benefit to atezolizumab in patients with higher PD-L1 expression. Nevertheless, there were no differences regarding OS according to this design and PD-L1 expression behaved as a prognostic rather than predictive factor, favouring also the chemotherapy arm. Yet, atezolizumab showed longer DoR, was better tolerated and was superior to chemotherapy in landmark analyses [17, 18].

We report data from an individual-level pooled analysis of the subset of Spanish patients in the IMvigor210 cohort 2 and IMvigor211 studies to explore whether their outcomes differed from overall results or not.

## Materials and methods

We analyzed the efficacy and safety of atezolizumab in Spanish patients that were recruited in the cohort 2 of the IMvigor 210 and IMvigor 211 studies. Patients of both studies were aged ≥ 18 years with advanced or metastatic urothelial carcinoma whose disease had progressed after previous platinum-based chemotherapy Baseline characteristics are presented in Table 1.

**Table 1:** Baseline characteristics

	ITT population (*n* = 131)	IC0/1 population (*n *= 98)	IC2/3 population (*n* = 33)	Atezolizumab (*n* = 74)	Chemotherapy (*n* = 57)
Age					
Median (years; range)	66 (41–85)	67.5 (41–85)	64.0 (4–83)	68.0 (41–85)	66.0 (47–84)
≥ 80 years	8 (6.1%)	6 (6.1%)	2 (6.1%)	7 (9.5%)	1 (1.8%)
Sex					
Female	23 (17.6%)	18 (18.4%)	5 (15.2%)	13 (17.6%)	10 (17.5%)
Male	108 (82.4%)	80 (81.6%)	28 (84.8%)	61 (82.4%)	47 (82.5%)
Tobacco use					
Current	21 (16.0%)	17 (17.3%)	4 (12.1%)	14 (18.9%)	7 (12.3%)
Former	26 (19.8%)	20 (20.4%)	6 (18.2%)	20 (27.0%)	6 (10.5%)
Never	84 (64.1%)	61 (62.2%)	23 (69.7%)	40 (54.1%)	44 (77.2%)
Primary tumour site					
Renal pelvis	12 (9.2%)	9 (9.2%)	3 (9.1%)	4 (5.4%)	8 (14.0%)
Ureter	17 (13.0%)	12 (12.2%)	5 (15.2%)	12 (16.2%)	5 (8.8%)
Bladder	100 (76.3%)	75 (76.5%)	25 (75.8%)	56 (75.7%)	44 (77.2%)
Urethra/Other	2 (1.5%)	2 (2.0%)	0 (0.0%)	2 (2.7%)	0
Metastatic disease	108 (82.4%)	82 (83.7%)	26 (78.8%)	64 (86.5%)	44 (77.2%)
Site of metastases					
Visceral	96 (73.3%)	73 (74.5%)	23 (69.7%)	59 (79.7%)	37 (64.9%)
Liver	32 (24.4%)	24 (24.5%)	8 (24.2%)	19 (25.7%)	13 (22.8%)
ECOG performance status					
0	53 (40.5%)	40 (40.8%)	13 (39.4%)	34 (45.9%)	19 (33.3%)
1	78 (59.5%)	58 (59.2%)	20 (60.6%)	40 (54.1%)	38 (66.6%)
Serum haemoglobin < 10 g/dl	14 (10.7%)	13 (13.3%)	1 (3.0%)	8 (10.8%)	6 (10.5%)
Number of risk factors					
0	40 (30.5%)	30 (30.6%)	10 (30.3%)	25 (33.8%)	15 (26.3%)
1	64 (48.9%)	46 (46.9%)	18 (54.5%)	34 (45.9%)	30 (52.6%)
2	21 (16.0%)	17 (17.3%)	4 (12.1%)	12 (16.2%)	9 (15.8%)
3	6 (4.6%)	5 (5.1%)	1 (3.0%)	3 (4.1%)	3 (5.3)
Previous cystectomy	62 (47.3%)	45 (45.9%)	17 (51.5%)	30 (40.5%)	32 (56.1%)
Time from previous chemotherapy < 3 months	43 (32.8%)	35 (35.7%)	8 (24.2%)	26 (35.1%)	17 (29.8%)
Number of previous systemic regimens in the metastatic setting					
0	43 (35.1%)	29 (29.6%)	14 (42.4%)	20 (27.0%)	23 (40.4%)
11	59 (45.0%)	48 (49.0%)	11 (33.3%)	34 (45.9%)	25 (43.9%)
2	23 (17.6%)	17 (17.3%)	6 (18.2%)	14 (18.9%)	15 (26.3%)
≥ 3	6 (4.6%)	4 (4.1%)	2 (6.1%)	6 (8.1%)	0
Previous systemic regimen setting					
Metastatic	88 (67.2%)	69 (70.4%)	19 (57.6%)	54 (73.0%)	34 (59.6%)
Neoadjuvant or adjuvant chemotherapy with progression within ≤ 12 months	42 (32.1%)	28 (28.6%)	14 (44.4%)	19 (25.7%)	23 (40.4%)
Other	1 (0.8%)	1 (1.0%)	0	1 (1.4%)	0

Data are median (range), *n* (%), or *n*/*N* (%), unless otherwise specified

Once data from both studies were combined, we described the ORR, DoR, PFS and OS, of patients who received atezolizumab (from both IMvigor210 and 211 studies) or chemotherapy (from IMvigor211), in the modified ITT population (all the subjects with their response assessed) and according to the PD-L1 expression (Ventana PD-L1 SP142 assay) on tumour-infiltrating immune cells (IC0/1 if < 5% of tumour-infiltrating immune expressed PD-L1 or IC2/3 if ≥ 5% expressed PD-L1). Safety outcomes were described in the ITT (all the randomized subjects).

Patients received atezolizumab 1200 mg or chemotherapy (vinflunine 320 mg/m^2^, paclitaxel 175 mg/m^2^ or docetaxel 75 mg/m^2^ based on the physician’s choice), intravenously every 3 weeks until unacceptable toxicity, RECIST v1.1 progression, or informed consent withdrawal.

## Results

Our sample comprised 21 (6.6%) patients out of the 315 recruited in the cohort 2 of the IMvigor 210 study plus 110 (11.8%) out of the 931 subjects in the IMvigor 211 study. These 131 patients treated in Spanish centers were assigned to either atezolizumab (*n* = 74; 56.5%) or chemotherapy (*n* = 57; 43.5%) and made up the intention-to-treat (ITT) population. However, the response was missing in 4 patients, and therefore the resulting 127 patients made up the modified ITT (mITT) population for efficacy outcomes, 74 (58.3%) on atezolizumab and 53 (41.7%) on chemotherapy (67.9% vinflunine and 32.1% taxanes).

**Fig. 1:**
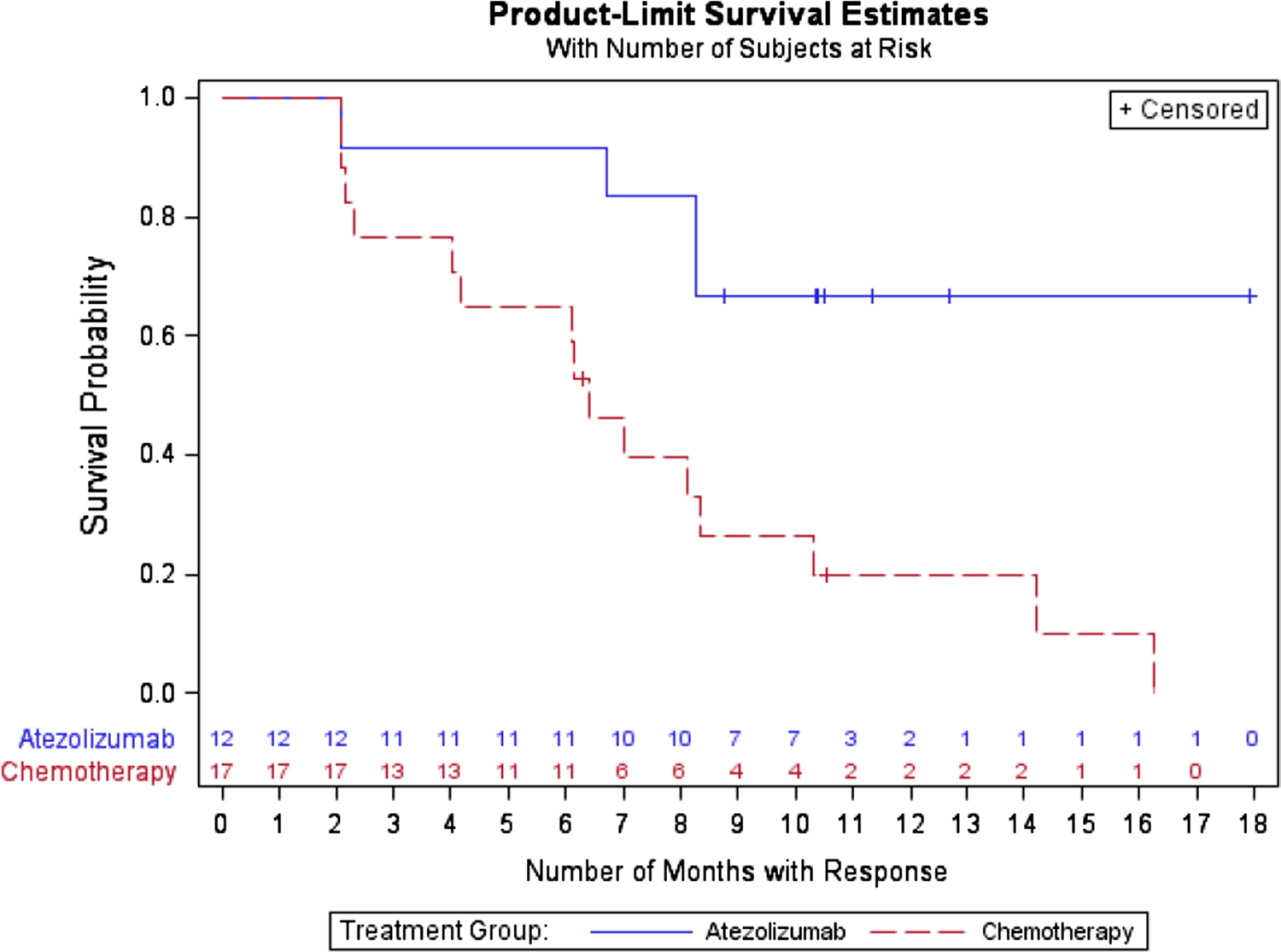
Duration of response in the mITT population. HR 0.24, 95% CI 0.07–0.66; *p* = 0.005

Analysis of the efficacy data revealed no statistically significant differences across arms in ORR with 12 (20.3%) responses out of the 59 evaluable patients in the atezolizumab arm compared with 17 (37.0%) out of the 46 patients in the chemotherapy arm (*p* = 0.059). Nonetheless, in those patients who responded, the DoR clearly favored the atezolizumab arm. Median DoR was not reached for atezolizumab vs 6.4 months for chemotherapy being these differences statistically significant (HR 0.24, 95% CI 0.07–0.66; *p* = 0.005) (Fig. 1). Moreover, remarkable differences were observed in indicators of long-term benefit. Among those patients who presented either a partial or complete response (12 and 17 patients in atezolizumab and chemotherapy, respectively), the 6-month survival rates were quite different in favor of the immunotherapy group. Thus, 91.7% of atezolizumab treated responders were alive at 6 months versus 64.5% of patients who received chemotherapy. This trend was maintained overtime and when analyzing the long-term efficacy, the differences between the two treatment arms were even larger. Hence, the 12 month survival rates were 66.7% and 19.9% for immunotherapy and chemotherapy patients, respectively.

Although the median progression-free survival (PFS) was 5.3 months (95% CI 3.7–6.7) in the chemotherapy arm compared to 2.1 months (95% CI 2.0–2.5) in the atezolizumab arm (p = 0.043), 12-month PFS rates were not significantly different (*p* = 0.0574), 12.2% (95% CI 5.0–22.8) for chemotherapy and 9.1% (95% CI 3.4–18.4) for atezolizumab.

**Fig. 2:**
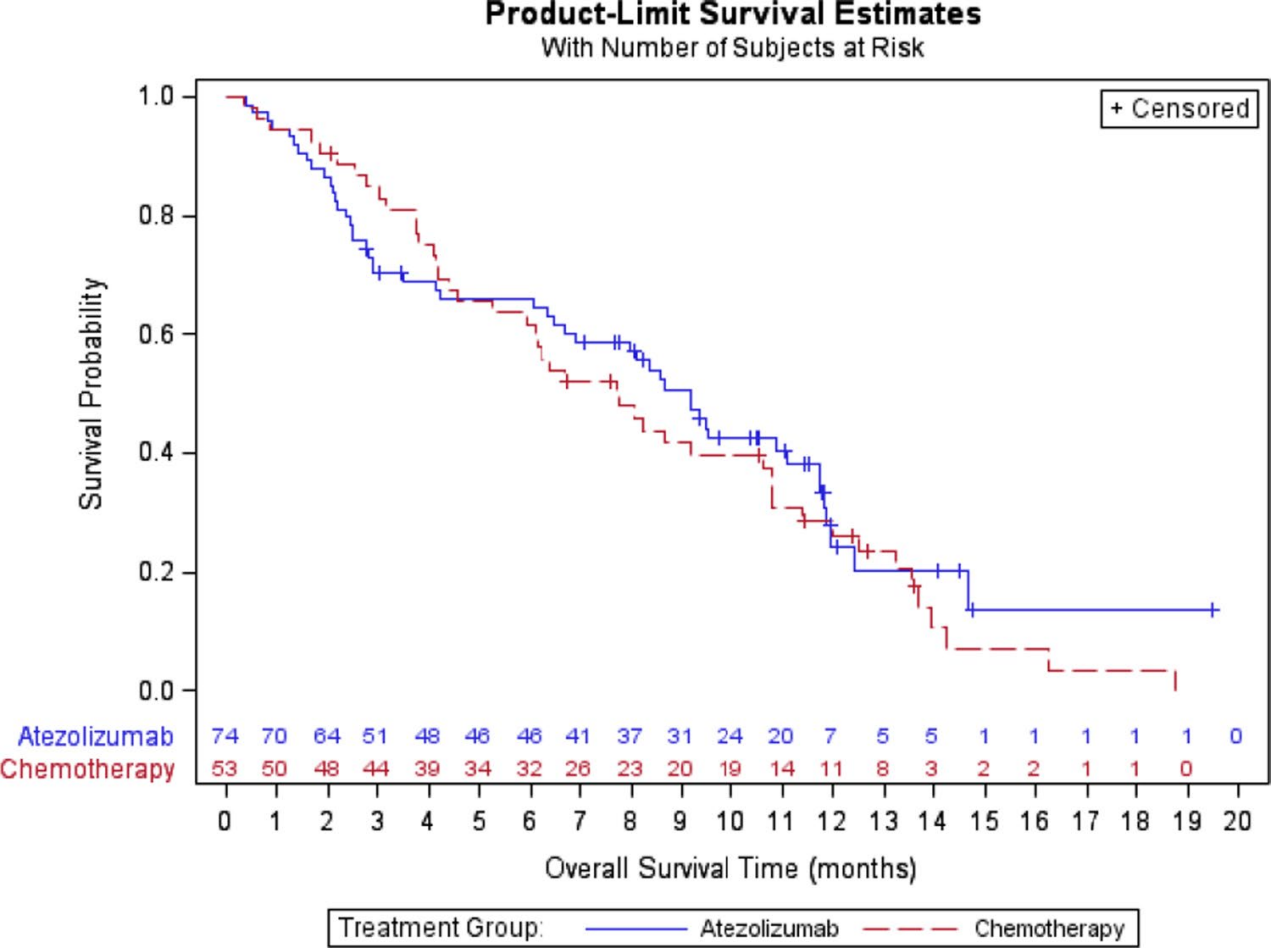
Overall survival in the mITT population. HR 0.86, 95% CI 0.57–1.29; *p* = 0.462

Median OS was numerically superior in the atezolizumab group compared to the chemotherapy group, (9.2 months; 95% CI 6.5–11.7 vs 7.7 months; 95% CI 5.3–10.8) albeit not reaching statistical significance (Fig. 2). However, patients in the atezolizumab group did have fewer events than in the chemotherapy group, (66.2% vs 84.9%; *p* = 0.018) Table 2.

Interestingly, when the efficacy analysis was performed stratifying patients according to the PD-L1 expression status on infiltrating immune cells (ICs) in the tumour microenvironment, our analyses did not find any significant difference either in ORR, DoR, PFS or OS regardless of the IC group in patients treated with either atezolizumab or chemotherapy (Table 2).

**Table 2:** Efficacy outcomes

PD-L1 score	mITT		IC0/1		IC2/3	
	Atezolizumab (*n* = 74)	Chemotherapy (*n* = 53)	Atezolizumab (*n* = 54)	Chemotherapy (*n* = 41)	Atezolizumab (*n* = 20)	Chemotherapy (*n* = 12)
Overall survival^a^						
Patients with event (%)	49 (66.2%)	45 (84.9%)	37 (68.5%)	36 (87.8%)	12 (60.0%)	9 (75.0%)
Median (months; 95% CI)	9.2 (6.5–11.7)	7.7 (5.3–10.8)	9.2 (6.5–11.7)	6.4 (4.1–8.6)	10.9 (2.4–12.4)	12.0 (4.2–13.6)
6 month overall survival rate (95% CI)	65.9% (53.8–75.5)	61.7% (47.1–73.3)	68.4% (54.2–79.0)	56.1% (39.7–69.6)	59.2% (34.7–77.2)	81.8% (44.7–95.1)
12 month overall survival rate (95% CI)	24.4% (13.1–37.6)	26.2% (14.8–39.1)	22.3% (10.5–36.9)	21.8% (10.4–35.9)	37.6% (15.2–60.1)	41.6% (13.1–68.4)
Progression-free survival^b^						
Patients with event (%)	67 (90.5%)	49 (92.5%)	51 (94.4%)	39 (95.1%)	16 (80.0%)	10 (83.3%)
Median (months; 95% CI)	2.1 (2.0–2.5)	5.3 (3.7–6.7)	2.1 (2.0–2.5)	4.2 (2.2–7.7)	2.2 (1.8–4.2)	5.3 (3.4–10.4)
12 month PFS rate (95% CI)	9.1% (3.4–18.4)	12.2% (5.0–22.8)	6.9% (2.0–16.1)	10.4% (3.3–22.1)	20.0% (6.2–39.3)	18.2% (2.9–44.2)
Objective response rate						
No. of evaluable patients	59	46	43	34	16	12
No. of patients with response (%; 95% CI)	12 (20.3%; 11.0–32.8)	17 (37.0%; 23.2–53.5)	7 (16.3%; 6.8–30.7)	12 (35.3%; 19.7–53.5)	5 (31.3%; 11.0–58.7)	5 (41.7%; 15.2–72.3)
Best overall response						
Complete response	3 (4.1%)	3 (5.7%)	1 (1.9%)	2 (4.9%)	2 (10.0%)	1 (8.3%)
Partial response	9 (12.2%)	14 (26.4%)	6 (11.1%)	10 (24.4%)	3 (15.0%)	4 (33.3%)
Stable disease	14 (18.9%)	18 (34.0%)	11 (20.4%)	11 (26.8%)	3 (15.0%)	7 (58.3%)
Progressive disease	33 (44.6%)	11 (20.8%)	25 (46.3%)	11 (26.8%)	8 (40.0%)	0
Missing or unevaluable	15 (20.3%)	7 (13.2%)	11 (20.4%)	7 (17.1%)	4 (20.0%)	0
Duration of response^c^						
Patients with event (%)	4 (33%)	15 (88%)	3 (42.9%)	11 (91.7%)	1 (20.0%)	4 (80.0%)
Median (months; 95% CI)	NE (6.7-NE)	6.4 (2.3–8.3)	NE (6.7-NE)	6.3 (2.2–10.3)	NA (2.1-NE)	7.0 (2.1-NE)
6 month survival rate (%; 95% CI)	91.7% (53.9–98.8)	64.7% (37.7–82.3)	100% (NE-NE)	66.7% (33.7–86.0)	80.0% (20.4–96.9)	60.0% (12.6–88.2)
12 month survival rate (%; 95% CI)	66.7% (33.7–86.0)	19.9% (3.3–34.3)	57.1% (17.2–83.5)	20.0% (3.3–46.9)	80.0% (20.4–96.9)	20% (0.8–58.2)

^a^Death

^b^Progressive disease or death

^c^Response stop or death

**Fig. 3:**
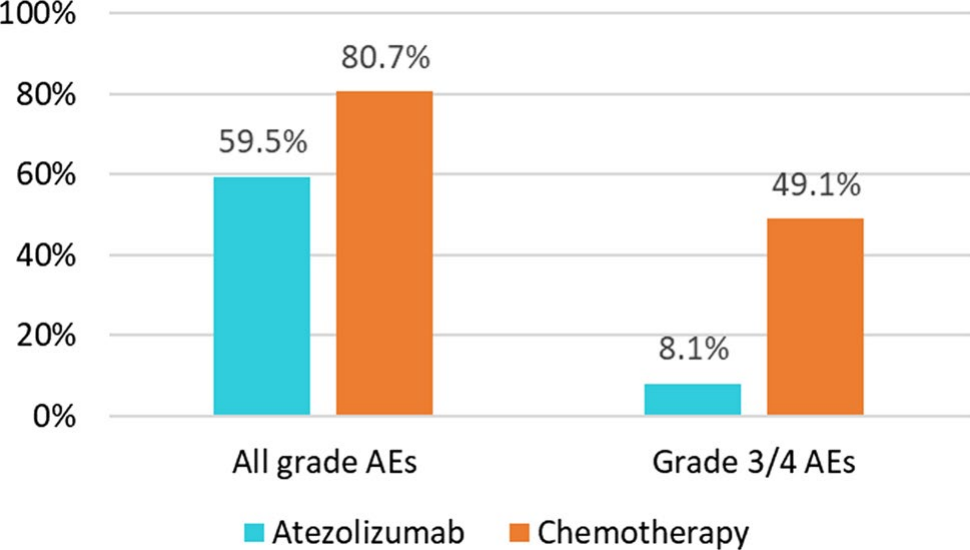
Safety in the ITT population. Proportion of patients with adverse events. Data are percentages of patients presenting with AEs

The safety analysis revealed that any-grade treatment-related adverse events occurred in 44/74 (59%) atezolizumab patients vs. 46/57 (81%) chemotherapy patients. Grade 3–4 adverse events were documented in 6 (8%) patients given atezolizumab vs. 28 (49%) given chemotherapy (Fig. 3). Among patients on chemotherapy, grade 3–4 AEs occurred in 57.5% of patients allocated to vinflunine and in 29% of patients assigned to taxanes.

The most frequent adverse events (those who affected to at least 10% of patients) were asthenia, pruritus and diarrhoea in patients treated with atezolizumab. For patients on chemotherapy, the commonest adverse events were asthenia, neutropenia, constipation, alopecia, abdominal pain, anaemia, nausea, diarrhoea, mucositis, decreased appetite and vomiting. The most common grade 3–4 adverse events in the chemotherapy group were neutropenia (28%), asthenia (12%), constipation (9%), anaemia (7%) and abdominal pain (5%). A detailed list of treatment-related AEis by frequency and severity is presented in Table 3.

**Table 3:** Treatment-related adverse events

	Atezolizumab (*n* = 74)	Chemotherapy (*n* = 57)
Most common treatment-related adverse events of any grade		
All	44 (59%)	46 (81%)
Asthenia	22 (30%)	28 (49%)
Pruritus	10 (14%)	1 (2%)
Diarrhoea	9 (12%)	8 (14%)
Decreased appetite	7 (10%)	7 (12%)
Rash	6 (8%)	2 (3%)
Arthralgia	4 (5%)	5 (9%)
Decreased neutrophil count	0	22 (39%)
Constipation	2 (3%)	21 (37%)
Alopecia	0	12 (21%)
Anemia	0	10 (18%)
Nausea	3 (4%)	10 (18%)
Mucosal inflammation	1 (1%)	8 (14%)
Vomiting	2 (3%)	7 (12%)
Abdominal pain	0	7 (12%)
Nail dystrophy	1 (1%)	5 (9%)
Decreased platelet count	0	5 (9%)
Dizziness	0	4 (7%)
Febrile neutropenia	0	3 (5%)
Increased lacrimation	0	3 (5%)
Myalgia	0	3 (5%)
Paraesthesia	0	3 (5%)
Pyrexia	1 (1%)	3 (5%)
Grade 3 or 4 treatment-related adverse events		
All	6 (8%)	28 (49%)
Asthenia	3 (4%)	7 (12%)
Ulcerative colitis	1 (1%)	0
Haematuria	1 (1%)	0
Intestinal obstruction	2 (3%)	2 (4%)
Constipation	0	5 (9%)
Decreased neutrophil count	0	16 (28%)
Anaemia	0	4 (7%)
Diaorrhea	0	1 (2%)
Abdominal pain	0	3 (5%)
Febrile neutropenia	0	3 (5%)
Peripheral neuropathy	0	1 (2%)
Phlebitis	0	1 (2%)
Sepsis	0	1 (2%)
Stomatitis	0	1 (2%)
Intestinal subocclusion	0	1 (2%)
General physical health deterioration	0	1 (2%)

Adverse events of all grades reported in at least 5% of patients in either group. All grade 3 or 4 are listed in both groups

## Discussion

For more than three decades, no progress has been made in advanced UC therapeutics [8]. Yet, the recent incorporation of immunotherapy with the arrival to the clinic of the check-point inhibitors has reshaped the treatment scenario, and both atezolizumab (an anti-PD-L1) and pembrolizumab (an anti-PD-1) have become standard options for patients with advanced UC with either progression beyond first-line chemotherapy or treatment naïve and unfit to receive cisplatin [19]. Immunotherapy has demonstrated substantial benefit in this patient population and a better toxicity profile across different trials [15, 16, 20, 21]. Nevertheless, there are still unanswered questions about the real impact and whether clinical or biological factors might help us predict the efficacy of this innovative treatment strategy or not [22]. Sub-analyses of certain populations in this context could unveil details relevant for a better understanding of the activity and safety and could help explain unexpected results or even pose new questions that might deserve to be answered. A pooled analysis of the Spanish patients in two relevant trials of atezolizumab in advanced UC patients is presented here. When analyzing efficacy, ORR showed no statistically significant differences between the immunotherapy and the chemotherapy arms as previously reported in other atezolizumab studies, such as IMvigor 211, where ORR was almost identical in both study arms [16]. Outcomes were slightly different with pembrolizumab when compared with chemotherapy in the Keynote-045 study where it achieved superior ORR (21.1% vs 11.4%; *p* = 0.001). Whether these results could be related to less use of vinflunine over taxanes in the Keynote-045 trial remains unanswered [21]. Another parameter of efficacy, such as OS, showed also no statistically significant differences between treatment groups in our analysis. Yet, the reported numerical values are consistent with the recently reported from real-world data studies of atezolizumab and the pivotal study of pembrolizumab [21, 23].

Furthermore, regarding the analysis of the predictive value of PD-L1 staining, no significant differences in OS were observed when patients were stratified according to this biomarker. A trend towards PD-L1 expression as a favourable prognostic factor was observed. Hence, those patients IC2/3 presented a better survival in both groups and actually, OS was superior in this subgroup for the chemotherapy arm although not reaching statistical significance. This adds to other reports that suggest that PD-L1 staining is a prognostic factor rather than predictive and could justify the lack of substantial differences in efficacy in these patient’s populations. Regarding PFS, the results obtained in this pooled analysis in favour of chemotherapy are overall consistent with most previous trials comparing cytotoxics with immunotherapy in different tumour types although PFS value as a surrogate marker of activity is questioned in this context [24]. Probably the most striking results of this work have to do with the long-term benefit observed in the responders in the immunotherapy group. For decades, one of the major limitations that has jeopardized progress of UC therapeutics has been the short duration of the responses presented to cytotoxic treatment. Despite relatively high ORR to chemotherapy in first line, virtually all responders will eventually relapse (most of the times early) and historical treatment options provided little or no benefit in this setting [25]. The results of our analysis illustrate remarkable differences in survival in the responders according to their treatment arm. Those patients who responded to atezolizumab presented a survival benefit at the 6 and 12 month landmarks. In the latter, around three times, more patients were alive in the atezolizumab responders compared to those who responded to chemotherapy. This behaviour appears common across different checkpoint inhibitors and different tumour types and is becoming one of the hallmarks of immunotherapy [26].

Lastly, our analysis comes to confirm the benefit of atezolizumab in terms of safety when compared with cytotoxics. As we already saw in IMvigor 211, the adverse-event profile of atezolizumab was also favourable compared with chemotherapy in the Spanish population.

The proportion of patients with any AE was lower in the atezolizumab treated patients as well as the severity of these events when compared with those who received chemotherapy. This confirms a distinctive common characteristic to most immunotherapy studies, the benefit of this treatment strategy in terms of safety, which results are particularly relevant in a patient population that normally present comorbidities. Despite the valuable information provided by these analyses, some limitations must be acknowledged, mainly the exploratory nature and the relatively low sample size despite combining two groups from two different studies.

Once more, as in the IMvigor 211 trial, PD-L1 expression does not seem to discriminate patients who might benefit from atezolizumab. Looking for biomarkers must be mandatory to identify patients with advanced urothelial cancer who might benefit from this immune checkpoint inhibitor.

In summary, this pooled analysis of the Spanish population included in the IMvigor210 cohort 2 and 211 trials corroborates the major hallmarks of immunotherapy. Despite not identifying statistically significant differences in ORR and OS and a worse PFS when compared with chemotherapy, atezolizumab demonstrated an unprecedented long-term benefit in those patients who responded to immunotherapy with a very favourable safety profile. Identifying potential predictive factors of response remains a critical unmet need.
